# Vaccination against COVID-19 in health care workers

**DOI:** 10.47626/1679-4435-2021-191

**Published:** 2021-04-30

**Authors:** Paula Cristina Moreira Couras da Silva

**Affiliations:** 1 Occupational Physician, Assistant Editor, Revista Brasileira de Medicina do Trabalho

Dear reader,

While COVID-19 has been a source of significant adversity, it has also allowed for advances in science and technology. The first wave of the pandemic was characterized by unfamiliarity. At that point, we did not know the virus binding site; its multisystemic effects, which extended far beyond the respiratory system; or any medication that could be used against it. We then embarked on a frantic search for a vaccine.

The second wave of the pandemic brought an increase in the number of cases relative to the first one. While older adults were still the most affected by the illness, the second wave of COVID-19 was brought about by individuals aged 20 to 49 years, who accounted for 60% of new cases and, by contributing to viral circulation, increased the likelihood of mutations.

Genetic variants of COVID-19, SARS-CoV-2, emerged, and the olfactory loss that had been among the most common symptoms of the illness at the start of the pandemic became less frequent. The most common symptoms in this new phase were muscle pain, sore throat, intense fatigue, fever, and cough. Since anosmia is not as common in patients infected with the new variant, people who have the illness may not recognize it as COVID-19, becoming less likely to seek health care services and increasing the spread of the virus.

The virus adapted to survive.

Nature is fantastic!

New variants of the virus are more infectious and some are more and others less lethal than the first version of SARS-CoV-2, placing greater demands on health care services, which may lead to an increase in mortality rates due to the collapse of the health care system. We must focus on prevention measures such as vaccinating health care workers, which is crucial for both prevention and health promotion purposes.

As occupational physicians, we have been on the front lines of the fight against COVID-19 with a high risk of exposure to SARS-CoV-2, as we never stopped treating workers of different occupational sectors with varying degrees of risk, in addition to providing assistance and support to patients with confirmed or suspected SARS-CoV-2 infection. We provide essential services on an uninterrupted basis, even if exhausted, afraid and unvaccinated.

Ensuring the health of workers in the health care sector should be a top priority, and organized vaccination program in primary care units should be set up, established for us, doctors, as we are the first line of defense against the pandemic, and as occupational doctors, as we are in the front-lines of the fight against COVID-19.

The health security of us health professionals is a top priority, and we need to be vaccinated with organized calls to vaccination posts, as we remain the first line of defense against this deadly pandemic. We, occupational physicians, must be at the top of this list, because we are front-line agents against COVID-19.

The Centers for Disease Control and Prevention recommend that health care workers be considered a priority group for vaccination, together with students in health care programs who are involved in the provision of care. This priority status should extend to all professionals doing paid or unpaid work in health care settings, where they are directly or indirectly exposed to infected patients, employees and/or materials. The vaccination of health care professionals ensures the continuity of health care services in the community.

The infection rate among health professionals is still high, at approximately 7.3% in contrast to 5% of the general population. Those who become ill and can no longer provide essential services to patients or workers are effectively barred from playing their important role in interpersonal care. As such, the protection of these professionals at work, at home and in the community should be a national priority.

The vaccination of health care teams contributes to the prevention of SARS-CoV-2, as it keeps the virus from circulating and undergoing further mutations. This is especially important in Brazil, a vast country with a large population whose movements are difficult to control. Health care workers infected with COVID-19 can also spread SARS-CoV-2 to patients, workers, colleagues, friends and relatives who are under their care and/or in their social circle. Many of these people may have comorbidities that increase the risk of severe illness or even death due to COVID-19.

The vaccines are safe and recommended to all age groups and risk groups. An unprotected population, on the other hand, poses a great risk to those who have not been vaccinated and anyone they may encounter. In addition to being immunized against COVID-19, occupational physicians should be up to date with their vaccinations, including those mandated by NR32 (tetanus-diphtheria and hepatitis B), influenza vaccines, and any additional immunizations recommended based on their region of residence and job (endemic) and age group, such as yellow fever, herpes zoster and pneumonia.

In conclusion, we are still far from knowing everything about SARS-CoV-2 and COVID-19, and we still have much to learn. We will also have to find effective ways to manage future waves of the pandemic, should they occur. Furthermore, we cannot ignore the possibility of future pandemics caused by other pathogens, and must think of ways to contain diseases with an aggressive clinical course while preventing the collapse of the health care system.

As occupational physicians, we will ever be in the front lines, but we should act with caution, aware that we are all vulnerable if unprotected. If we are ill, we hurt the workforce. If vaccinated, we are strong, and can continue to work with our usual motivation and ethical commitment.

We hope you have a good read!

